# Supplementary material for: Quantitative Systems Pharmacological Analysis of Drugs of Abuse Reveals the Pleiotropy of Their Targets and the Effector Role of mTORC1
Source: Front Pharmacol. 2019 Mar 8;10:191. doi: 10.3389/fphar.2019.00191 (PMC6418047; doi:10.3389/fphar.2019.00191)
Supplement: Supplementary file 1 [file Table_1.docx]

Supplementary Material

*for*

Quantitative systems pharmacological analysis of drugs of abuse reveals the pleiotropy of their targets and the effector role of mTORC1

Fen Pei^†^, Hongchun Li^†^, Bing Liu^*^ and Ivet Bahar^*^

*Department of Computational and Systems Biology, School of Medicine,*

*University of Pittsburgh, PA, 15213, USA*

**^*^ Correspondence:** Bing Liu liubing@pitt.edu and Ivet Bahar: bahar@pitt.edu

**^†^** These authors made equal contributions

# Supplementary Figures and Tables

## Supplementary Figures

| 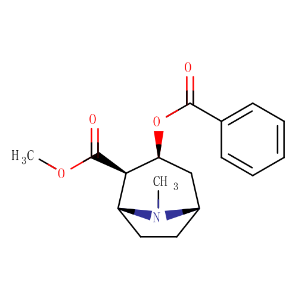 Cocaine | 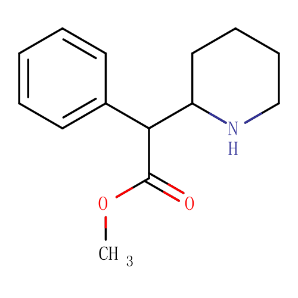 Methylphenidate | 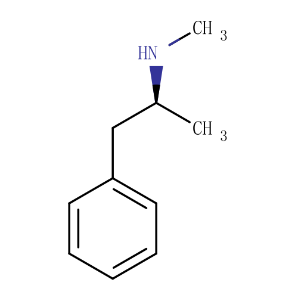 Methamphetamine | 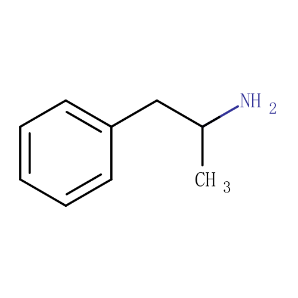 Amphetamine |
| --- | --- | --- | --- |
| 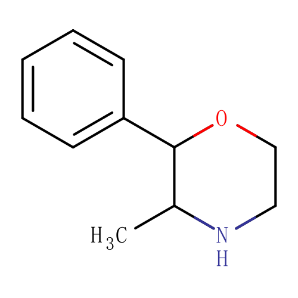 Phenmetrazine | 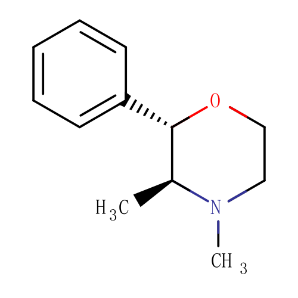 Phendimetrazine | 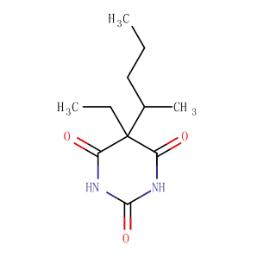 Pentobarbital | 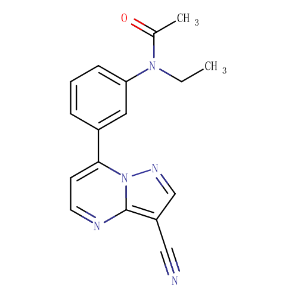 Zaleplon |
| 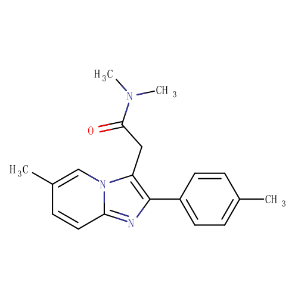 Zolpidem | 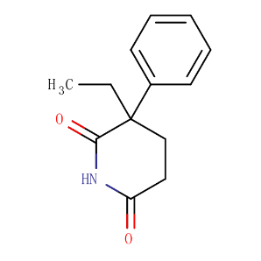 Glutethimide | 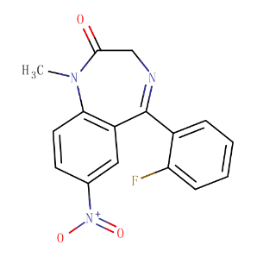 Flunitrazepam | 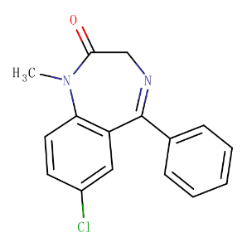 Diazepam |
| 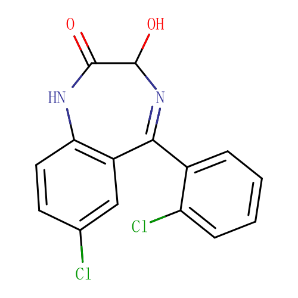 Lorazepam | 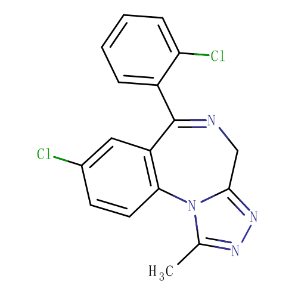 Triazolam | 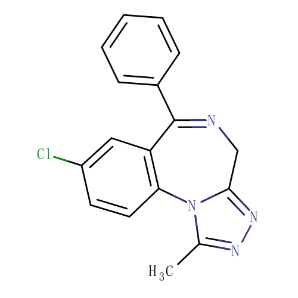 Alprazolam | 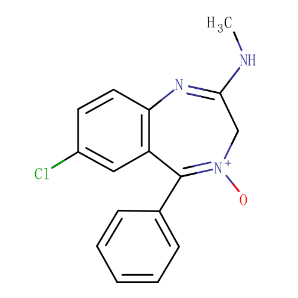 Chlordiazepoxide |
| 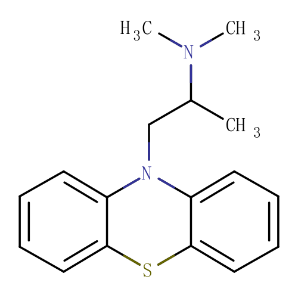 Promethazine | 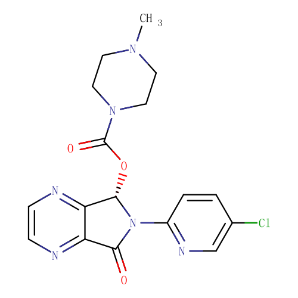 Eszopiclone | 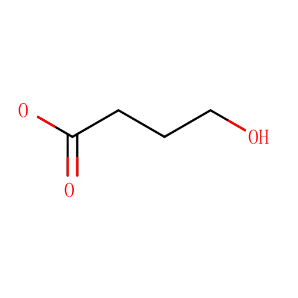 Gamma Hydroxybutyric Acid (GHB) | 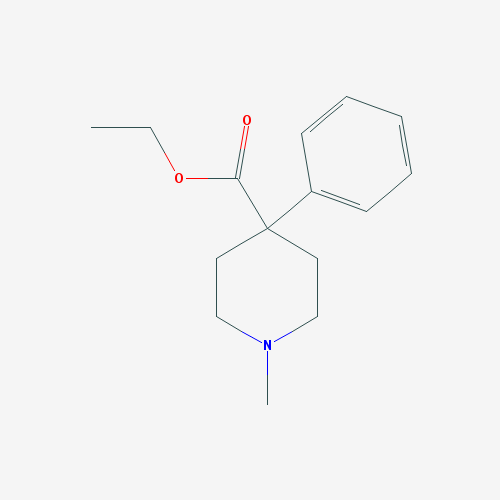  Meperidine |
| 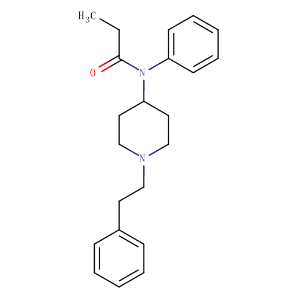 Fentanyl | 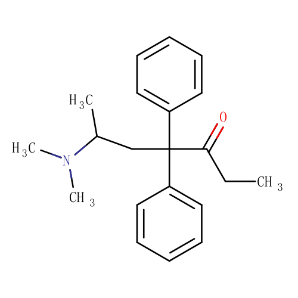 Methadone | 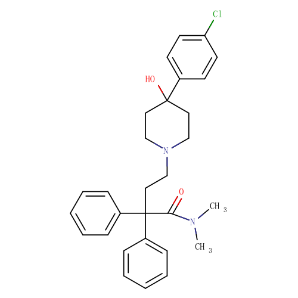 Loperamide | 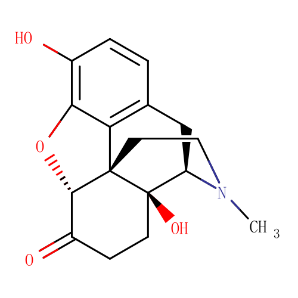 Oxymorphone |
| 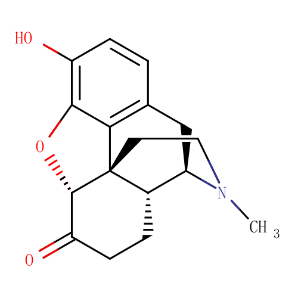 Hydromorphone | 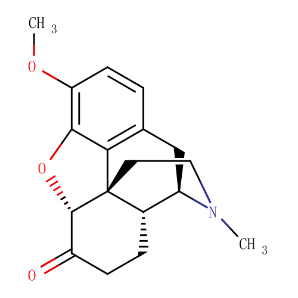 Hydrocodone | 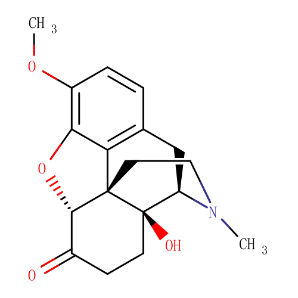 Oxycodone | 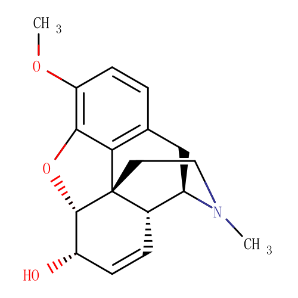 Codeine |
| 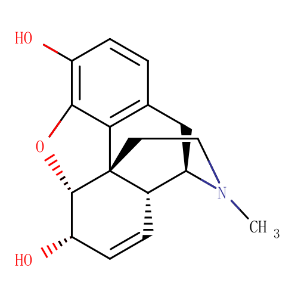 Morphine | 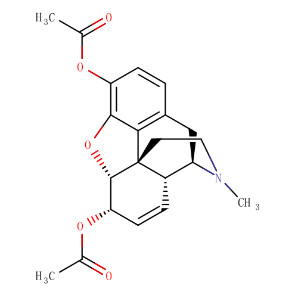 Heroin | 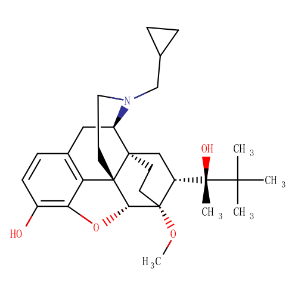 Buprenorphine | 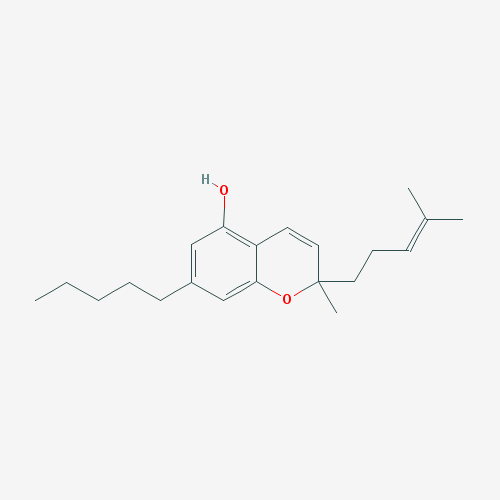 Cannabichromene |
| 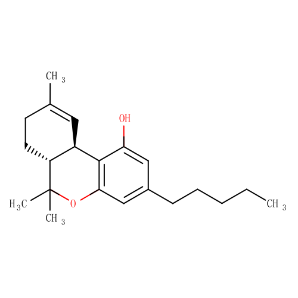 Dronabinol | 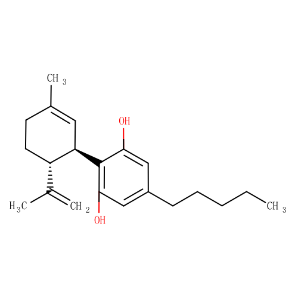 Cannabidiol | 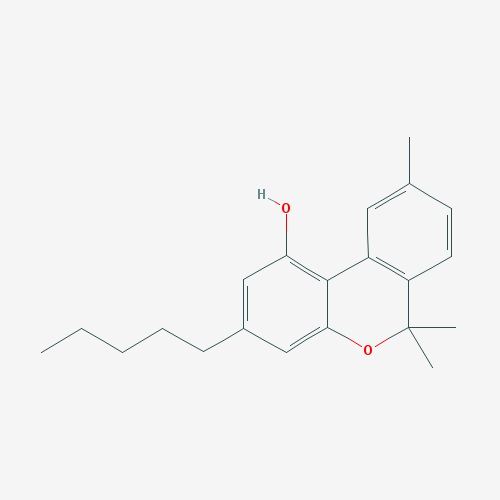 Cannabinol | 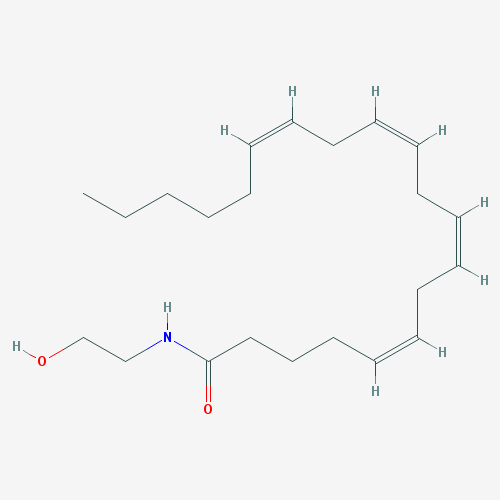 Anandamide |
| 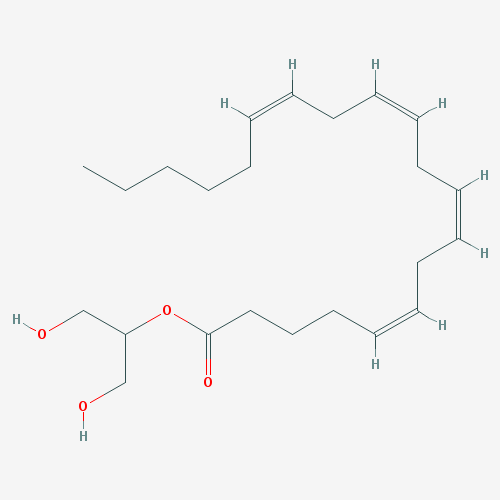 2-AG | 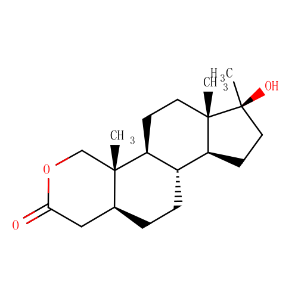 Oxandrolone | 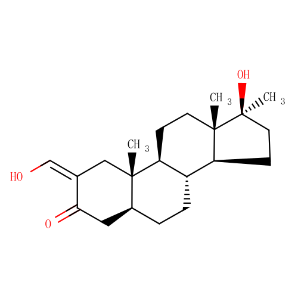 Oxymetholone | 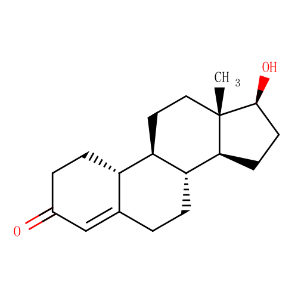 Nandrolone |
| 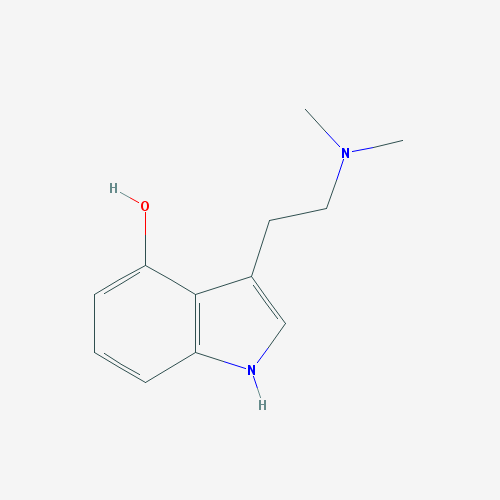 Psilocin | 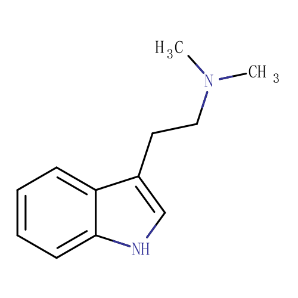 Dimethyltryptamine | 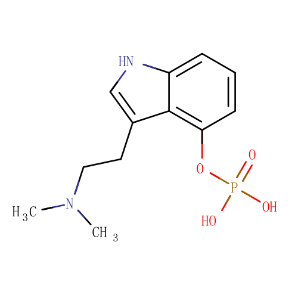 Psilocybin | 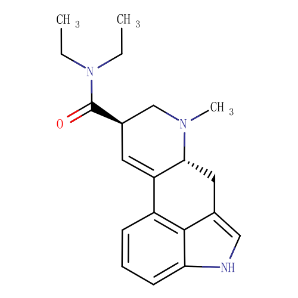 Lysergic Acid Diethylamide (LSD) |
| 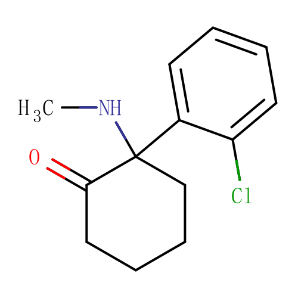 Ketamine | 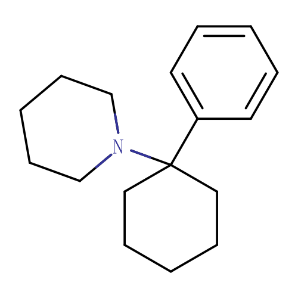 Phencyclidine | 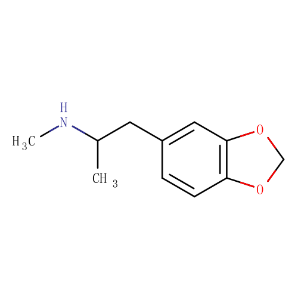 Midomafetamine | 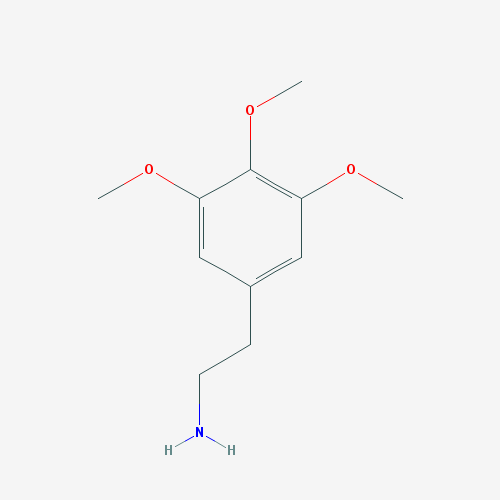 Mescaline |
| 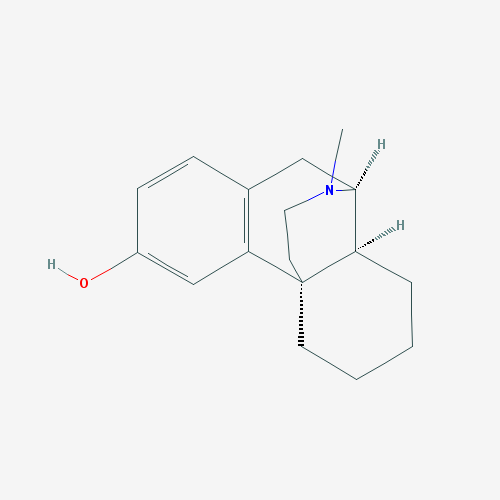 Dextrorphan | 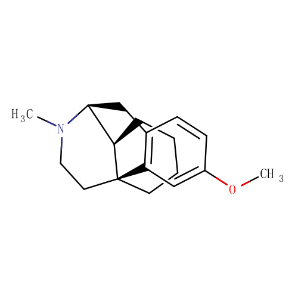 Dextromethorphan |  |  |

**Supplementary Figure 1. 2D structures of the 50 addictive drugs listed in Supplementary Table 1.** The names of drugs/chemicals are colored *green*, *blue*, red, *cyan*, *light brown*, *black* and *magenta* for the 6 CNS stimulants, 13 CNS depressants, 12 opioids, 7 cannabinoids, 4 anabolic steroids and 10 hallucinogens, respectively.

**Supplementary Figure 2. Prediction of new targets for known drugs of abuse. (A)** Drug-target interactions are shown for three drugs of abuse, cocaine, cannabichromene and nandrolone. The colors of the drug nodes are consistent with the label colors in **Figure 2C**; the diamond nodes represent targets, the color of the target nodes is consistent with the label colors in **Figure 2F**; diamonds nodes with *red* borders represent predicted targets, diamond nodes without borders are the known targets; *blue* edges are known interactions and *red* edges are predicted interactions. Note that the norepinephrine transporter (NET/SLC6A2) and dopamine transporter (DAT/SLC6A3) are shared between cocaine and cannabichromene. Corticotropin-releasing factor receptor 1 (CRHR1) was predicted to be a new target shared between cannabichromene and nandrolone. See the full names of the known and predicted targets in the respective **Supplementary Tables 2** and **3**. **(B)** Drug-target interactions are shown for opioid receptors. Green diamonds are opioid receptors (OPRs): OPRM1, OPRD1 and OPRK1; 12 opioids interact with three OPRs either by existing evidence or prediction; hallucinogens ketamine and dextromethorphan also interact with three OPRs; a novel interaction between OPRM1 and the CNS stimulant methylphenidate was predicted.


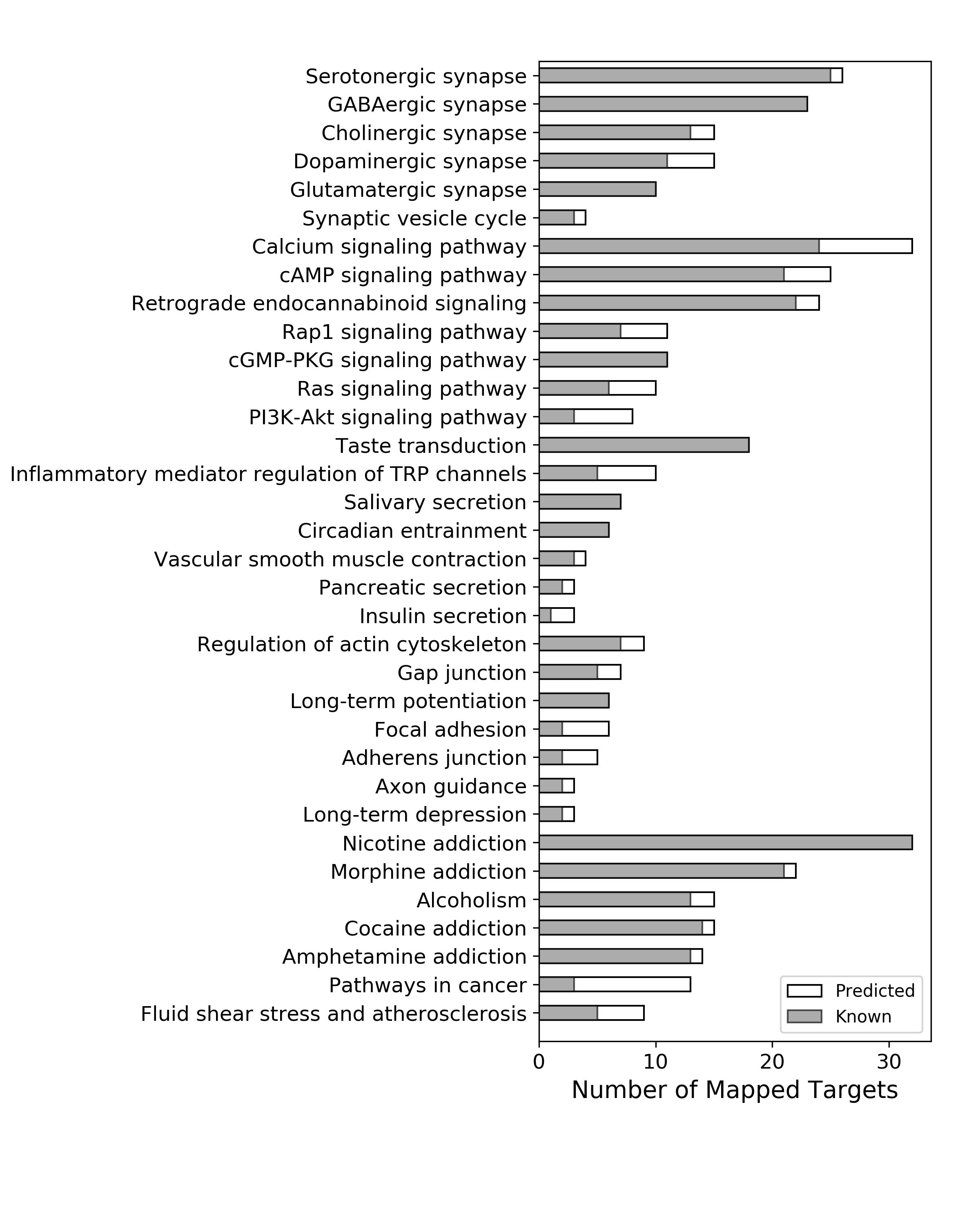


**Supplementary Figure 3. Pathways distinguished by high numbers of targets associated with drugs of abuse, organized in five categories, as listed in Figure 4.** The pathways listed in **Supplementary Table 4** are ordered by the *p*-value calculated by merged targets. The bars here represent the number of known (*gray*) and predicted (*white*) proteins targeted by drugs of abuse in each pathway. Up to seven pathways have been included in each case.

**Supplementary Figure 4. Pathway and target enrichments in five functional categories and the overlap of targets in different categories. (A)** Numbers of drug addiction pathways (*red*) and targets (*gray*) in the five pathway categories (NT: neurotransmission related pathways; SG: signal transduction pathways; DS: disease pathways; ANS: ANS-innervation related pathways and NP: neuroplasticity related pathways) identified from known (*left*) targets and predicted targets (*right*) exclude those pathways and targets involved in known drug-target interactions, respectively. (**B**) Numbers of overlapping known (*left*) and predicted (*right*) targets between NT, DS and SG pathway categories. (**C**) Overlap results as in (**B**), between NT, SG and NP pathway categories. (**D**) Overlap results as in (**B**), between NT, ANS and NP pathway categories. See detailed information on the identities of pathways and targets corresponding to the five functional categories in the **Supplementary Table 4**.


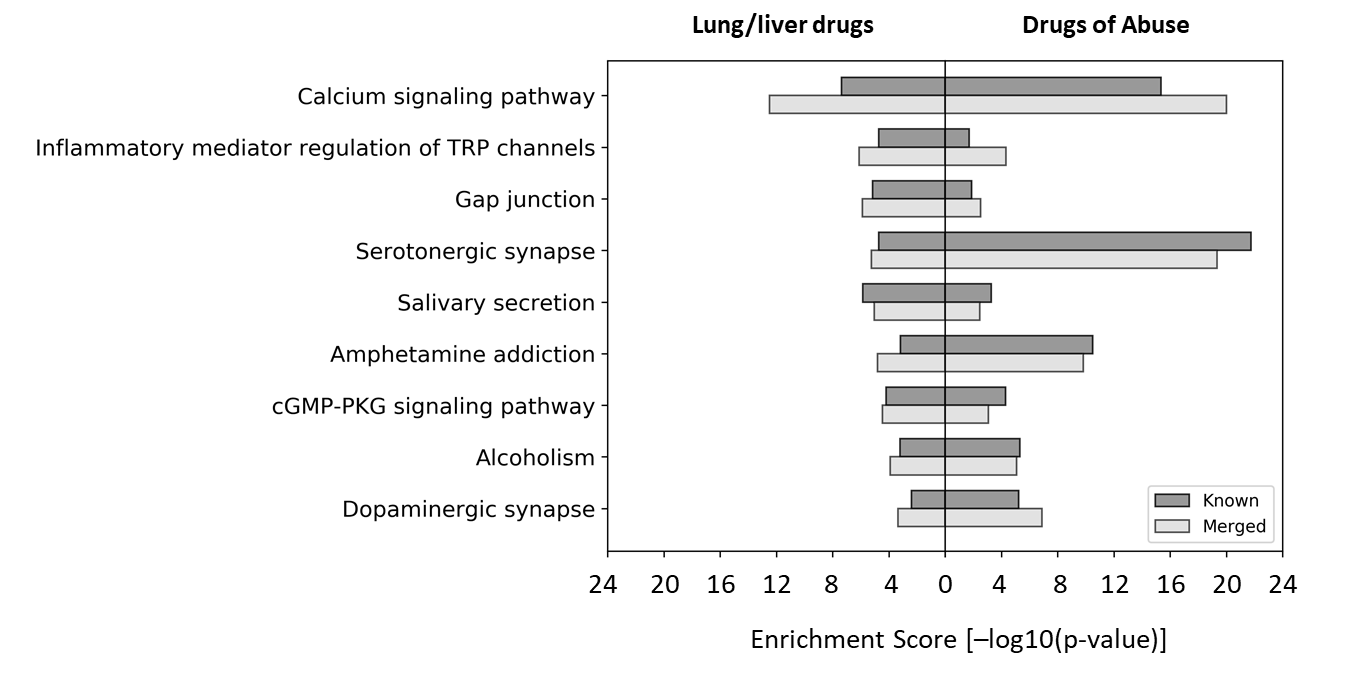


**Supplementary Figure 5**. **The** **enriched overlapping pathways for lung/liver drugs and drugs of abuse.** Enrichment *p*-values based on known (*gray*) or merged (*light gray*) targets (including both known and predicted targets) are calculated separately for each pathway, the pathways are ranked by the *p*-value calculated by merged targets of lung/liver drugs listed in **Supplementary Table 7**. There are 10 overlapping pathways with enrichment score (-log10(p-value)) over 2, between the pathways enriched by the examined sets of lung/liver drugs and drugs of abuse. Note that the neuroactive ligand-receptor interaction pathway, which has the highest enrichment score (known: 16.8, merged: 6.5 for lung/liver drugs; known: 104.6, merged: 93.05 for drugs of abuse) is not shown for visualization purpose.

## Supplementary Tables

**Supplementary Table 1. 50 addictive drugs and their corresponding groups and identifiers**

| **Index** | **Drug group and name** | | **DrugBank ID** | **Pubchem ID** | **# of targets** | **Reference** |
| --- | --- | --- | --- | --- | --- | --- |
| **1** | **CNS Stimulants** | Cocaine | DB00907 | 446220 | 45 | (Gawin and Ellinwood, 1988) |
| 2 |  | Methylphenidate | DB00422 | 4158 | 3 | (Klein-Schwartz, 2002) |
| 3 |  | Methamphetamine | DB01577 | 10836 | 11 | (Winslow et al., 2007) |
| 4 |  | Amphetamine | DB00182 | 3007 | 17 | (Kramer et al., 1967) |
| 5 |  | Phenmetrazine | DB00830 | 4762 | 2 | (Mellar and Hollister, 1982) |
| 6 |  | Phendimetrazine | DB01579 | 30487 | 3 | (Bolin et al., 2016) |
| 7 | **CNS Depressants** | Pentobarbital | DB00312 | 4737 | 27 | (Griffiths et al., 1979) |
| 8 |  | Zaleplon | DB00962 | 5719 | 2 | (Dooley and Plosker, 2000) |
| 9 |  | Zolpidem | DB00425 | 5732 | 13 | (Madrak and Rosenberg, 2001) |
| 10 |  | Glutethimide | DB01437 | 3487 | 16 | (Jones and Mayberry, 1986) |
| 11 |  | Flunitrazepam | DB01544 | 3380 | 16 | (Druid et al., 2001) |
| 12 |  | Diazepam | DB00829 | 3016 | 24 | (Woody et al., 1975) |
| 13 |  | Lorazepam | DB00186 | 3958 | 17 | (Troisi 2nd et al., 1993) |
| 14 |  | Triazolam | DB00897 | 5556 | 20 | (Fleming, 1983) |
| 15 |  | Alprazolam | DB00404 | 2118 | 17 | (Rush et al., 1993) |
| 16 |  | Chlordiazepoxide | DB00475 | 2712 | 16 | (Hollister et al., 1961) |
| 17 |  | Promethazine | DB01069 | 4927 | 19 | (Tsay et al., 2015) |
| 18 |  | Eszopiclone | DB00402 | 969472 | 17 | (Hajak et al., 2003) |
| 19 |  | Gamma Hydroxybutyric Acid (GHB) | DB01440 | 11266 | 2 | (Galloway et al., 2000) |
| 20 | **Opioids** | Meperidine | NA | 4058 | 2 | (Joranson et al., 2000) |
| 21 |  | Fentanyl | DB00813 | 3345 | 3 | (Gold et al., 2006) |
| 22 |  | Methadone | DB00333 | 4095 | 11 | (Cicero and Inciardi, 2005) |
| 23 |  | Loperamide | DB00836 | 3955 | 5 | (Lasoff et al., 2017) |
| 24 |  | Oxymorphone | DB01192 | 5284604 | 3 | (Babalonis et al., 2016) |
| 25 |  | Hydromorphone | DB00327 | 5284570 | 3 | (Walsh et al., 2008) |
| 26 |  | Hydrocodone | DB00956 | 5284569 | 2 | (Babalonis et al., 2016) |
| 27 |  | Oxycodone | DB00497 | 5284603 | 5 | (Harris et al., 2014) |
| 28 |  | Codeine | DB00318 | 5284371 | 3 | (Kathiramalainathan et al., 2000) |
| 29 |  | Morphine | DB00295 | 5288826 | 4 | (Preston et al., 1991) |
| 30 |  | Heroin | DB01452 | 5462328 | 3 | (Büttner et al., 2000) |
| 31 |  | Buprenorphine | DB00921 | 644073 | 4 | (Oconnor et al., 1988) |
| 32 | **Cannabinoids** | Cannabichromene | NA | 30219 | 1 | (Poklis et al., 2010) |
| 33 |  | Dronabinol | DB00470 | 16078 | 3 | (Calhoun et al., 1998) |
| 34 |  | Cannabidiol | DB09061 | 644019 | 2 | (Robson, 2011) |
| 35 |  | Cannabinol | NA | 2543 | 2 | (Yamamoto et al., 2003) |
| 36 |  | Anandamide | NA | 5281969 | 2 | (Solinas et al., 2007) |
| 37 |  | 2-AG | NA | 5282280 | 2 | (Solinas et al., 2007) |
| 38 | **Steroids** | Oxandrolone | DB00621 | 5878 | 1 | (Bahrke and Yesalis, 2004) |
| 39 |  | Oxymetholone | DB06412 | 5281034 | 2 | (Bahrke and Yesalis, 2004) |
| 40 |  | Nandrolone | DB13169 | 9904 | 2 | (Kouvelas et al., 2008) |
| 41 | **Hallucinogens** | Psilocin | NA | 4980 | 11 | (Ludwig and Levine, 1965) |
| 42 |  | Dimethyltryptamine | DB01488 | 8441 | 3 | (Winstock et al., 2014) |
| 43 |  | Psilocybin | DB11664 | 10624 | 5 | (Passie et al., 2002) |
| 44 |  | Lysergic Acid Diethylamide (LSD) | DB04829 | 5761 | 12 | (Simpson et al., 1997) |
| 45 |  | Ketamine | DB01221 | 3821 | 20 | (Dotson et al., 1995) |
| 46 |  | Phencyclidine | DB03575 | 6468 | 10 | (Slavney et al., 1977) |
| 47 |  | Midomafetamine | DB01454 | 1615 | 8 | (Seger, 2010) |
| 48 |  | Mescaline | NA | 4076 | 2 | (Neiman et al., 2000) |
| 49 |  | Dextrorphan | NA | 5360697 | 1 | (Schwartz, 2005) |
| 50 |  | Dextromethorphan | DB00514 | 5360696 | 21 | (Boyer, 2004) |

‘# of targets’ counts the number of targets of drugs recorded in both DrugBank v5 (Wishart et al., 2018) and STITCH v5 (Szklarczyk et al., 2016).

**Supplementary Tables 2-4** and **6** are presented in Excel files.

| **Supplementary Table 5. Enrichment of the 190 targets of addictive drugs in the brain** | | | | | |
| --- | --- | --- | --- | --- | --- |
| **Category^(a)^** | **Count** | | **Ref^(b)^** | **ER^(c)^** | **Targets^(d)^** |
| Elevated in brain | 49 | 1460 | | **3.4%** | **HTR5A; GABRB1; GRIA2; GABRG2; GABRG1; CHRNB2; GRIN2B; HTR2A; HTR2C; SLC6A17; GABRA5; GABRA4; GRIN1; GABRD; GABRA1; GABRB2; GABRA3; GABRA2; HRH3; P2RY12**; SLC6A1; SLC6A7; OPRL1; CNR1; CACNA1A; GRIN3A; SLC6A11; SLC6A15; CHRM5; CHRM4; CHRM3; CHRM1; CHRNA4; OPRK1; GABRB3; ADRA1B; GRIK2; GABRQ; GRIN2C; GRIN2A; HTR3B; OPRD1; GRIN2D; HTR1A; CCKBR; GLP1R; DRD5; CRHR1; DRD1 |
| Moderately expressed in brain | 82 | 13058 | | 0.6% | SLC52A2; RAC2; PGRMC1; RAC1; CHRNB1; GABRP; CYBA; BRD4; PRCP; TSPO; SIGMAR1; ERBB2; NR3C1; HDAC6; S1PR1; EPHX2; MAPK14; PPARD; HMGCR; CTSS; CDK2; NR1H2; DPP7; NCF2; NCF1; SLC6A6; NCF4; GABRG3; SLC6A9; SLC6A8; ADRB2; BCHE; ADRB1; TMIGD3; ADRA2A; ADRA2C; HRH1; SLC6A13; SLC6A12; SLC6A16; CHRM2; CYBB; HTR7; AR; CARTPT; CHRNA2; POMC; ACHE; TACR1; ADRA1A; SLC6A20; ADRA1D; KCNH2; ALB; MAOB; MAOA; CHRNA7; HTR1B; GABRE; HTR1E; HTR1F; CHRNA5; PTGS2; IGF1R; PTGDR2; CRHR2; CALCRL; DHFR; PIK3CA; EGFR; THRB; IKBKB; PPARG; DHFR2; PTAFR; TYMS; SRD5A1; TRPV2; TRPV1; F10; P2RX7; CHEK1 |
| Not detected in brain | 59 | 5095 | | 1.2% | CHRNE; SLC6A2; SLC6A5; SLC6A4; GABRR1; CHRNA10; AOX1; GPR55; TRPA1; GABRA6; CNR2; CHRNB3; CHRNB4; ADRA2B; XDH; SLC6A19; SLC6A18; PGR; HTR2B; NPPB; SLC6A14; SCN11A; HTR3E; GRIN3B; OPRM1; CHRNG; CHRNA1; CHRNA3; CHRNA9; TAAR1; ORM1; ORM2; DRD2; DRD3; ADRB3; HTR3C; CHRND; HTR3A; HTR1D; SLC18A2; SLC18A1; SCN5A; VDR; ESR2; CCKAR; GCGR; TRPV4; CALCA; SLC6A3; GABRR2; GABRR3; HRH4; SCN10A; HTR6; HTR3D; CHRNA6; SLC18A3; GLRA1; DRD4 |
| Total | 190 | 19613 | |  |  |
| *^(a)^Categories of targets (genes) are defined based on the mRNA expression levels of genes with the unit of Transcript Per Million (TPM) (Uhlén et al., 2015). Elevated in brain: > 5-fold higher than other tissues (targets in bold are the most enriched in the brain); Moderately expressed in brain: at least 1 TPM in brain and other tissues; Not detected in brain: less than 1 TPM in brain.  ^(b)^Number of genes in human proteome belong to each category. ^(c)^Enrichment ratio (ER) is the ratio of between numbers in Count and Ref columns. The ER in category of “Elevated in brain” is ~5-fold higher than that in “Moderately expressed in brain”.  ^(d)^Predicted targets are colored in red.* | | | | | |

**Supplementary Table 7. 15 autophagy-modulating drugs^(a)^ for liver and lung diseases.**

| **No.** | **Drug name** | **DrugBank ID** | **Pubchem ID** | **Disease** | **Reference** |
| --- | --- | --- | --- | --- | --- |
| **1** | Carbamazepine | DB00564 | 2554 | alpha1-antitrypsin deficiency; hepatic fibrosis; lung Proteinopathy | (Hidvegi et al., 2010; Hidvegi et al., 2015) |
| 2 | Fluphenazine | DB00623 | 3372 | alpha1-antitrypsin deficiency; lung Proteinopathy | (Li et al., 2014; Hidvegi et al., 2015) |
| 3 | Cantharidin | NA | 5944 | alpha1-antitrypsin deficiency | (Krichevsky et al., 2010) |
| 4 | Pimozide | DB01100 | 16362 | alpha1-antitrypsin deficiency | (Park et al., 2010) |
| 5 | Tamoxifen | DB00675 | 2733525 | alpha1-antitrypsin deficiency | (De Mol et al., 2010) |
| 6 | Phenylbutyric Acid | NA | 4775 | alpha1-antitrypsin deficiency | (Burrows et al., 2000) |
| 7 | Vorinostat | NA | 5311 | alpha1-antitrypsin deficiency | (Bouchecareilh et al., 2012) |
| 8 | Glycerol | DB09462 | 753 | alpha1-antitrypsin deficiency | (Burrows et al., 2000) |
| 9 | Fluspirilene | DB04842 | 3396 | alpha1-antitrypsin deficiency | (O'reilly et al., 2014) |
| 10 | Ezetimibe | NA | 150311 | alpha1-antitrypsin deficiency | (Yamamura et al., 2014) |
| 11 | Gemfibrozil | DB01241 | 3463 | COPD-emphysema | (Bodas et al., 2017a) |
| 12 | Fisetin | NA | 5281614 | COPD-emphysema | (Bodas et al., 2017a) |
| 13 | Cysteamine | NA | 6058 | COPD-emphysema | (Bodas et al., 2016; Shivalingappa et al., 2016) |
| 14 | S-Nitrosoglutathione | NA | 104858 | COPD-emphysema | (Bodas et al., 2017b) |
| 15 | Rapamycin | NA | 5284616 | lung fibrosis; cystic fibrosis | (Kouvelas et al., 2008; Abdulrahman et al., 2011; Patel et al., 2012) |

*^(a)^These drugs are known autophagy modulators and have been reported to have therapeutic effects for liver and lung diseases.*

# Supplementary References

Abdulrahman, B.A., Khweek, A.A., Akhter, A., Caution, K., Kotrange, S., Abdelaziz, D.H., et al. (2011). Autophagy stimulation by rapamycin suppresses lung inflammation and infection by Burkholderia cenocepacia in a model of cystic fibrosis. *Autophagy* 7(11)**,** 1359-1370. doi: 10.4161/auto.7.11.17660.

Babalonis, S., Lofwall, M.R., Nuzzo, P.A., and Walsh, S.L. (2016). Pharmacodynamic effects of oral oxymorphone: abuse liability, analgesic profile and direct physiologic effects in humans. *Addict. Biol.* 21(1)**,** 146-158. doi: 10.1111/adb.12173.

Bahrke, M.S., and Yesalis, C.E. (2004). Abuse of anabolic androgenic steroids and related substances in sport and exercise. *Curr. Opin. Pharm.* 4(6)**,** 614-620.

Bodas, M., Patel, N., Silverberg, D., Walworth, K., and Vij, N. (2017a). Master autophagy regulator transcription factor EB regulates cigarette smoke-induced autophagy impairment and chronic obstructive pulmonary disease–emphysema pathogenesis. *Antioxid. Redox Signal.* 27(3)**,** 150-167.

Bodas, M., Silverberg, D., Walworth, K., Brucia, K., and Vij, N. (2017b). Augmentation of S-nitrosoglutathione controls cigarette smoke-induced inflammatory–oxidative stress and chronic obstructive pulmonary disease-emphysema pathogenesis by restoring cystic fibrosis transmembrane conductance regulator function. *Antioxid. Redox Signal.* 27(7)**,** 433-451.

Bodas, M., Van Westphal, C., Carpenter-Thompson, R., Mohanty, D.K., and Vij, N. (2016). Nicotine exposure induces bronchial epithelial cell apoptosis and senescence via ROS mediated autophagy-impairment. *Free Radical Biol. Med.* 97**,** 441-453.

Bolin, B.L., Stoops, W.W., Sites, J.P., and Rush, C.R. (2016). Abuse Potential of Oral Phendimetrazine in Cocaine-dependent Individuals: Implications for Agonist-like Replacement Therapy. *J. Addict. Med.* 10(3)**,** 156-165. doi: 10.1097/ADM.0000000000000206.

Bouchecareilh, M., Hutt, D.M., Szajner, P., Flotte, T.R., and Balch, W.E. (2012). Histone deacetylase inhibitor (HDACi) suberoylanilide hydroxamic acid (SAHA)-mediated correction of α1-antitrypsin deficiency. *J. Biol. Chem.* 287(45)**,** 38265-38278.

Boyer, E.W. (2004). Dextromethorphan abuse. *Pediatr. Emerg. Care* 20(12)**,** 858-863.

Burrows, J.A., Willis, L.K., and Perlmutter, D.H. (2000). Chemical chaperones mediate increased secretion of mutant α1-antitrypsin (α1-AT) Z: a potential pharmacological strategy for prevention of liver injury and emphysema in α1-AT deficiency. *PNAS* 97(4)**,** 1796-1801.

Büttner, A., Mall, G., Penning, R., and Weis, S. (2000). The neuropathology of heroin abuse. *Forensic Sci. Int.* 113(1-3)**,** 435-442.

Calhoun, S.R., Galloway, G.P., and Smith, D.E. (1998). Abuse potential of dronabinol (Marinol). *J. Psychoactive Drugs* 30(2)**,** 187-196. doi: 10.1080/02791072.1998.10399689.

Cicero, T.J., and Inciardi, J.A. (2005). Diversion and abuse of methadone prescribed for pain management. *JAMA* 293(3)**,** 293-298. doi: 10.1001/jama.293.3.297.

De Mol, P., Krabbe, H.G., De Vries, S.T., Fokkert, M.J., Dikkeschei, B.D., Rienks, R., et al. (2010). Accuracy of handheld blood glucose meters at high altitude. *PLoS One* 5(11)**,** e15485.

Dooley, M., and Plosker, G.L. (2000). Zaleplon. *Drugs* 60(2)**,** 413-445. doi: 10.2165/00003495-200060020-00014.

Dotson, J.W., Ackerman, D.L., and West, L.J. (1995). Ketamine Abuse. *J. Drug Iss.* 25(4)**,** 751-757. doi: Doi 10.1177/002204269502500407.

Druid, H., Holmgren, P., and Ahlner, J. (2001). Flunitrazepam: an evaluation of use, abuse and toxicity. *Forensic Sci. Int.* 122(2-3)**,** 136-141.

Fleming, J.A. (1983). Triazolam abuse. *Can. Med. Assoc. J.* 129(4)**,** 324-325.

Galloway, G., Frederick-Osborne, S., Seymour, R., Contini, S.E., and Smith, D.E. (2000). Abuse and therapeutic potential of gamma-hydroxybutyric acid. *Alcohol* 20(3)**,** 263-269.

Gawin, F.H., and Ellinwood, E.H., Jr. (1988). Cocaine and other stimulants. Actions, abuse, and treatment. *N. Engl. J. Med.* 318(18)**,** 1173-1182. doi: 10.1056/NEJM198805053181806.

Gold, M.S., Melker, R.J., Dennis, D.M., Morey, T.E., Bajpai, L.K., Pomm, R., et al. (2006). Fentanyl abuse and dependence: further evidence for second hand exposure hypothesis. *J. Addict. Dis.* 25(1)**,** 15-21. doi: 10.1300/J069v25n01_04.

Griffiths, R.R., Bigelow, G., and Liebson, I. (1979). Human drug self-administration: double-blind comparison of pentobarbital, diazepam, chlorpromazine and placebo. *J. Pharmacol. Exp. Ther.* 210(2)**,** 301-310.

Hajak, G., Müller, W.E., Wittchen, H.U., Pittrow, D., and Kirch, W. (2003). Abuse and dependence potential for the non-benzodiazepine hypnotics zolpidem and zopiclone: a review of case reports and epidemiological data. *Addiction* 98(10)**,** 1371-1378. doi: doi:10.1046/j.1360-0443.2003.00491.x.

Harris, S.C., Perrino, P.J., Smith, I., Shram, M.J., Colucci, S.V., Bartlett, C., et al. (2014). Abuse potential, pharmacokinetics, pharmacodynamics, and safety of intranasally administered crushed oxycodone HCl abuse-deterrent controlled-release tablets in recreational opioid users. *J. Clin. Pharmacol.* 54(4)**,** 468-477. doi: 10.1002/jcph.235.

Hidvegi, T., Ewing, M., Hale, P., Dippold, C., Beckett, C., Kemp, C., et al. (2010). An autophagy-enhancing drug promotes degradation of mutant α1-antitrypsin Z and reduces hepatic fibrosis. *Science* 329(5988)**,** 229-232.

Hidvegi, T., Stolz, D.B., Alcorn, J.F., Yousem, S.A., Wang, J., Leme, A.S., et al. (2015). Enhancing autophagy with drugs or lung-directed gene therapy reverses the pathological effects of respiratory epithelial cell proteinopathy. *J. Biol. Chem.* 290(50)**,** 29742-29757. doi: 10.1074/jbc.M115.691253.

Hollister, L.E., Motzenbecker, F.P., and Degan, R.O. (1961). Withdrawal reactions from chlordiazepoxide (“Librium”). *Psychopharmacologia* 2(1)**,** 63-68. doi: 10.1007/bf00429621.

Jones, A.H., and Mayberry, J.F. (1986). Chronic glutethimide abuse. *Br. J. Clin. Pract.* 40(5)**,** 213.

Joranson, D.E., Ryan, K.M., Gilson, A.M., and Dahl, J.L. (2000). Trends in medical use and abuse of opioid analgesics. *JAMA* 283(13)**,** 1710-1714. doi: 10.1001/jama.283.13.1710.

Kathiramalainathan, K., Kaplan, H.L., Romach, M.K., Busto, U.E., Li, N.Y., Sawe, J., et al. (2000). Inhibition of cytochrome P450 2D6 modifies codeine abuse liability. *J. Clin. Psychopharmacol.* 20(4)**,** 435-444.

Klein-Schwartz, W. (2002). Abuse and toxicity of methylphenidate. *Curr. Opin. Pediatr.* 14(2)**,** 219-223.

Kouvelas, D., Pourzitaki, C., Papazisis, G., Dagklis, T., Dimou, K., and Kraus, M.M. (2008). Nandrolone abuse decreases anxiety and impairs memory in rats via central androgenic receptors. *Int. J. Neuropsychopharmacol.* 11(7)**,** 925-934. doi: 10.1017/S1461145708008754.

Kramer, J.C., Fischman, V.S., and Littlefield, D.C. (1967). Amphetamine abuse: Pattern and effects of high doses taken intravenously. *JAMA* 201(5)**,** 305-309. doi: 10.1001/jama.1967.03130050039011.

Krichevsky, A., Meyers, B., Vainstein, A., Maliga, P., and Citovsky, V. (2010). Autoluminescent plants. *PLoS One* 5(11)**,** e15461.

Lasoff, D.R., Koh, C.H., Corbett, B., Minns, A.B., and Cantrell, F.L. (2017). Loperamide Trends in Abuse and Misuse Over 13 Years: 2002-2015. *Pharmacotherapy* 37(2)**,** 249-253. doi: 10.1002/phar.1885.

LESLIE N. MADRAK, and MARK ROSENBERG (2001). Zolpidem Abuse. *Am. J. Psychiatry* 158(8)**,** 1330-a-1331. doi: 10.1176/appi.ajp.158.8.1330-a.

Li, J., Pak, S.C., O’Reilly, L.P., Benson, J.A., Wang, Y., Hidvegi, T., et al. (2014). Fluphenazine reduces proteotoxicity in C. elegans and mammalian models of alpha-1-antitrypsin deficiency. *PLoS One* 9(1)**,** e87260.

Ludwig, A.M., and Levine, J. (1965). Patterns of hallucinogenic drug abuse. *JAMA* 191(2)**,** 92-96. doi: 10.1001/jama.1965.03080020020006.

Mellar, J., and Hollister, L.E. (1982). Phenmetrazine: an obsolete problem drug. *Clin. Pharmacol. Ther.* 32(6)**,** 671-675.

Neiman, J., Haapaniemi, H.M., and Hillbom, M. (2000). Neurological complications of drug abuse: pathophysiological mechanisms. *Eur. J. Neurol.* 7(6)**,** 595-606.

O'reilly, L.P., Long, O.S., Cobanoglu, M.C., Benson, J.A., Luke, C.J., Miedel, M.T., et al. (2014). A genome-wide RNAi screen identifies potential drug targets in a C. elegans model of α1-antitrypsin deficiency. *Hum. Mol. Genet.* 23(19)**,** 5123-5132.

Oconnor, J.J., Moloney, E., Travers, R., and Campbell, A. (1988). Buprenorphine Abuse among Opiate Addicts. *Br. J. Addict.* 83(9)**,** 1085-1087.

Park, J., Jeon, Y., In, D., Fishel, R., Ban, C., and Lee, J.-B. (2010). Single-molecule analysis reveals the kinetics and physiological relevance of MutL-ssDNA binding. *PLoS One* 5(11)**,** e15496.

Passie, T., Seifert, J., Schneider, U., and Emrich, H.M. (2002). The pharmacology of psilocybin. *Addict. Biol.* 7(4)**,** 357-364. doi: 10.1080/1355621021000005937.

Patel, A.S., Lin, L., Geyer, A., Haspel, J.A., An, C.H., Cao, J., et al. (2012). Autophagy in idiopathic pulmonary fibrosis. *PLoS One* 7(7)**,** e41394. doi: 10.1371/journal.pone.0041394.

Poklis, J.L., Thompson, C.C., Long, K.A., Lichtman, A.H., and Poklis, A. (2010). Disposition of cannabichromene, cannabidiol, and Delta(9)-tetrahydrocannabinol and its metabolites in mouse brain following marijuana inhalation determined by high-performance liquid chromatography-tandem mass spectrometry. *J. Anal. Toxicol.* 34(8)**,** 516-520.

Preston, K.L., Jasinski, D.R., and Testa, M. (1991). Abuse potential and pharmacological comparison of tramadol and morphine. *Drug Alcohol Depend.* 27(1)**,** 7-17.

Robson, P. (2011). Abuse potential and psychoactive effects of δ-9-tetrahydrocannabinol and cannabidiol oromucosal spray (Sativex), a new cannabinoid medicine. *Expert Opinion on Drug Safety* 10(5)**,** 675-685. doi: 10.1517/14740338.2011.575778.

Rush, C.R., Higgins, S.T., Bickel, W.K., and Hughes, J.R. (1993). Abuse liability of alprazolam relative to other commonly used benzodiazepines: a review. *Neurosci. Biobehav. Rev.* 17(3)**,** 277-285.

Schwartz, R.H. (2005). Adolescent Abuse of Dextromethorphan. *Clinical Pediatrics* 44(7)**,** 565-568. doi: 10.1177/000992280504400702.

Seger, D. (2010). Cocaine, metamfetamine, and MDMA abuse: the role and clinical importance of neuroadaptation. *Clinl Toxicol* 48(7)**,** 695-708. doi: 10.3109/15563650.2010.516263.

Shivalingappa, P.C., Hole, R., Van Westphal, C., and Vij, N. (2016). Airway exposure to e-cigarette vapors impairs autophagy and induces aggresome formation. *Antioxid. Redox Signal.* 24(4)**,** 186-204.

Simpson, D., Braithwaite, R.A., Jarvie, D.R., Stewart, M.J., Walker, S., Watson, I.W., et al. (1997). Screening for drugs of abuse (II): Cannabinoids, lysergic acid diethylamide, buprenorphine, methadone, barbiturates, benzodiazepines and other drugs. *Ann. Clin. Biochem.* 34 ( Pt 5)**,** 460-510. doi: 10.1177/000456329703400502.

Slavney, P.R., Rich, G.B., Pearlson, G.D., and McHugh, P.R. (1977). Phencyclidine abuse and symptomatic mania. *Biol. Psychiatry* 12(5)**,** 697-700.

Solinas, M., Yasar, S., and Goldberg, S.R. (2007). Endocannabinoid system involvement in brain reward processes related to drug abuse. *Pharmacol. Res.* 56(5)**,** 393-405.

Szklarczyk, D., Santos, A., von Mering, C., Jensen, L.J., Bork, P., and Kuhn, M. (2016). STITCH 5: augmenting protein-chemical interaction networks with tissue and affinity data. *Nucleic Acids Res.* 44(D1)**,** D380-384. doi: 10.1093/nar/gkv1277.

Troisi 2nd, J.R., Critchfield, T.S., and Griffiths, R.R. (1993). Buspirone and lorazepam abuse liability in humans: behavioral effects, subjective effects and choice. *Behav. Pharmacol.* 4(3)**,** 217-230.

Tsay, M.E., Procopio, G., Anderson, B.D., and Klein-Schwartz, W. (2015). Abuse and Intentional Misuse of Promethazine Reported to US Poison Centers: 2002 to 2012. *J. Addict. Med.* 9(3)**,** 233-237. doi: 10.1097/ADM.0000000000000124.

Uhlén, M., Fagerberg, L., Hallström, B.M., Lindskog, C., Oksvold, P., Mardinoglu, A., et al. (2015). Tissue-based map of the human proteome. *Science* 347(6220). doi: 10.1126/science.1260419.

Walsh, S.L., Nuzzo, P.A., Lofwall, M.R., and Holtman, J.R., Jr. (2008). The relative abuse liability of oral oxycodone, hydrocodone and hydromorphone assessed in prescription opioid abusers. *Drug Alcohol Depend.* 98(3)**,** 191-202. doi: 10.1016/j.drugalcdep.2008.05.007.

Winslow, B.T., Voorhees, K.I., and Pehl, K.A. (2007). Methamphetamine abuse. *American family physician* 76(8)**,** 1169-1174.

Winstock, A.R., Kaar, S., and Borschmann, R. (2014). Dimethyltryptamine (DMT): prevalence, user characteristics and abuse liability in a large global sample. *J Psychopharmacol* 28(1)**,** 49-54. doi: 10.1177/0269881113513852.

Wishart, D.S., Feunang, Y.D., Guo, A.C., Lo, E.J., Marcu, A., Grant, J.R., et al. (2018). DrugBank 5.0: a major update to the DrugBank database for 2018. *Nucleic Acids Res.* 46(D1)**,** D1074-D1082. doi: 10.1093/nar/gkx1037.

Woody, G.E., O'Brien, C.P., and Greenstein, R. (1975). Misuse and abuse of diazepam: an increasingly common medical problem. *Int. J. Addict.* 10(5)**,** 843-848.

Yamamoto, I., Watanabe, K., Matsunaga, T., Kimura, T., Funahashi, T., and Yoshimura, H. (2003). Pharmacology and toxicology of major constituents of marijuana - On the metabolic activation of cannabinoids and its mechanism. *J Toxicol-Toxin Rev* 22(4)**,** 577-589. doi: 10.1081/Txr-120026915.

Yamamura, T., Ohsaki, Y., Suzuki, M., Shinohara, Y., Tatematsu, T., Cheng, J., et al. (2014). Inhibition of Niemann-Pick-type C1-like1 by ezetimibe activates autophagy in human hepatocytes and reduces mutant alpha1-antitrypsin Z deposition. *Hepatology* 59(4)**,** 1591-1599. doi: 10.1002/hep.26930.
